# p53 inhibition attenuates cisplatin-induced acute kidney injury through microRNA-142-5p regulating SIRT7/NF-κB

**DOI:** 10.1080/0886022X.2022.2039195

**Published:** 2022-02-28

**Authors:** Guoxiao Chen, Huanzhou Xue, Xiangsheng Zhang, Degang Ding, Shilong Zhang

**Affiliations:** a Department of Urology, Zhengzhou University People’s Hospital, Henan Provincial People’s Hospital, Zhengzhou, China;; b Department of Surgery, Zhengzhou University People’s Hospital, Henan Provincial People’s Hospital, Zhengzhou, China

**Keywords:** Acute kidney injury, cisplatin, miR-142-5p, p53

## Abstract

Renal tubular epithelial cell apoptosis is the main mechanism of cisplatin-induced acute kidney injury. The role of microRNAs (miRNAs) in the apoptosis of renal tubular epithelial cells has been suggested, but the underlying mechanism has not been fully elucidated. We used microarray analysis to identify miR-142-5p involved in cisplatin-induced acute kidney injury. miR-142-5p was down-regulated in human renal tubular epithelial (HK-2) cells with cisplatin treatment. Notably, the overexpression of miR-142-5p attenuated the cisplatin-induced HK-2 cell apoptosis and inhibition of miR-142-5p aggravated cisplatin-induced HK-2 cell apoptosis. During cisplatin treatment, p53 was activated. The inhibition of p53 by pifithrin-α attenuated the cisplatin-induced kidney injury and up-regulated miR-142-5p expression. We also identified the Sirtuin7 (SIRT7) as a target of miR-142-5p. Furthermore, we demonstrated that the inhibition of SIRT7 prevented cisplatin-induced HK-2 cell apoptosis and decreased the expression of nuclear factor kappa B (NF-κB). Our data revealed that p53 inhibition could attenuate cisplatin-induced acute kidney injury by up-regulating miR-142-5p to repress SIRT7/NF-κB. These findings may provide a novel therapeutic target of cisplatin-induced acute kidney injury.

## Introduction

Acute kidney injury (AKI) causes 2 million deaths per year worldwide [1]. At present, no specific therapy has been developed to prevent or treat AKI, and various potential drugs have been recently identified and tested. Cisplatin is one of the most widely used broad-spectrum anti-cancer drugs in clinical practice and major causes of clinical AKI [2]. The nephrotoxicity of cisplatin restricts its clinical use [3,4], because its accumulation in the kidney leads to AKI, especially in the renal tubules [5]. Renal tubular injury involves several different mechanisms, such as apoptosis [6], autophagy [7], activation of MAPK signaling pathway [8], direct toxicity to renal tubular epithelial cells [9], DNA damage [10], and mitochondrial dysfunction [11]. An increasing number of studies has shown that apoptosis is the main mechanism of cisplatin-induced renal tubular epithelial cell injury. Current clinical treatment mainly involves hydration, diuretics, and dehydrating agents for the prevention of cisplatin-induced renal damage. However, considering the large amount of liquid required and the slow onset of effect, this condition may lead to disorder of water electrolyte and acid-base balance and increased the cardiac load [12,13]. Therefore, the mechanism of cisplatin in renal tubular epithelial cells should be studied, and the effective prevention and treatment of AKI should be explored.

microRNAs (miRNAs) are endogenous miRNA molecules composed of 19–25 nucleotides and plays an important role in gene expression regulation [14]. miRNAs are usually combined with partially complementary sequences in the 3′-untranslated region (3′-UTR) of the target gene to inhibit the expression of the target gene [15].

miRNAs contribute to cell development, differentiation, proliferation, apoptosis, and metabolism [16]. Many clinical and experimental animal studies have shown that microRNA plays an important role in kidney development and the pathogenesis of various diseases [17]. However, the mechanism in which specific miRNAs mediate renal tubular epithelial cells injury in cisplatin nephrotoxicity is unknown. We have analyzed miRNA change in the kidney during cisplatin treatment by microarray analysis, and the results show that miR-142-5p is a key miRNA induced by p53. In this paper, we further explored the mechanism of p53 regulating miR-142-5p in cisplatin-induced acute renal injury.

## Material and methods

### Cisplatin-induced acute kidney injury mouse model

Six- to eight-week-old male C57 mice were obtained from Cavens Animal Center of Changzhou (Changzhou, China). During the experiment, the animals were free to drink water and eat standard feed. Ventilation, lighting, temperature, and humidity were well maintained. After 7 days of adaption, the mice were provided with an intraperitoneal injection of 30 mg/kg cisplatin and euthanized at 1 or 2 days after injection, while the control group was injected with the same amount of saline. Moreover, mice were treated with miR-142-5p agomir (Ruibo, Guangzhou, China) and subjected to cisplatin. In the experiment to test the effects of pifithrin-α, pifithrin-α was mixed with cisplatin solution for intraperitoneal injection in mice. The dose of pifithrin-α (2.2 mg/kg) was chosen accordingly to a previous study [16]. One side of the kidney was fixed in 4% paraformaldehyde solution for paraffin section, while the other half was quickly transferred to liquid nitrogen and stored in a refrigerator at 80 °C on the next day for further experiment. All procedures were approved by Zhengzhou University People’s Hospital (no. 2019-0037) and complied with the Guidelines for the Care and Use of Laboratory Animals.

### Apoptosis model of renal tubular epithelial cells induced by cisplatin

The human renal proximal tubular epithelial (HK‐2) cells were cultured in Dulbecco’s modified Eagle’s medium (DMEM) supplemented with 10% fetal bovine serum (Gibco, USA). The culture medium was changed every 1–2 days and passaged by trypsin every 4–5 days. The cells in logarithmic growth phase were digested and inoculated in a six-well plate. After the cells were fused to 70%–80%, the cells were treated with 10 μM cisplatin (Sigma, USA) for 24 and 48 h. HK-2 cells were treated with or without Pifithrin-a (10 μM) accordingly to a previous study [17].

### Renal function and histology assay

The blood urea nitrogen (BUN) and serum creatinine were examined using commercial kits from Nanjing Jiancheng (Nanjing, China). Paraffin-embedded tissue specimens were continuously sectioned (4 μm in thickness), dried at 37° C for 12 h, and then stained with hematoxylin-eosin (HE) and Periodic Acid-Schiff (PAS) staining. The morphological damage showed necrosis of tubular epithelial cells, loss of brush border, and tubule expansion. According to the proportion of the damaged tubules in the total renal tubules, the scores were allocated as follows: no lesion was 0 point; <25% was 1 point; 25%–50% was 2 points; 50%–75% was 3 points; and >75% was 4 points. The pathological changes of renal specimens were observed under light microscope.

### Immunohistochemical and immunofluorescence staining

Paraffin-embedded mouse kidney sections were prepared *via* a routine procedure. Immunohistochemical staining and immunofluorescence staining were performed in accordance with routine protocols. Tissue sections were incubated with primary antibody p53 (1:150), caspase-3 (1:400), and E-cadherin (1:400) overnight at 4 °C. Sections were washed with PBS and incubated with secondary antibody at room temperature for 1 h.

### Cell transfection

MiR-142-5p mimic and anti-miR-142-5p-LNA were synthesized by Guangzhou Ruibo Co., Ltd. (Guangzhou, China), and SIRT7 siRNA was obtained from Shanghai GeneChem (Shanghai, China). MiR-142-5p mimic, anti-miR-142-5p-LNA, and SIRT7 siRNA were transfected into cells at the optimal concentration in accordance with manufacturer's instructions. Western blot analysis was carried out to examine the SIRT7 RNA inference efficacy, and PCR was used to confirm the efficiency of miR-142-5p mimics and anti-miR-142-5p-LNA.

### RNA sequencing

Total RNA was extracted, and its integrity was detected by agarose gel electrophoresis. Its purity, concentration, and integrity of total RNA were also detected by ABLife Co., Ltd (Wuhan, China). The total RNA was reverse transcribed and amplified by Smart-Seq2 method. After purification, 1 µl of the product was obtained, and its concentration and integrity were detected using Agilent 2100. The amplified products were used for library construction and sequencing.

### Luciferase reporter assay

The SIRT7 3′-UTR carrying a miR-142-5p binding site was constructed using PCR and subsequently cloned into the pMIR-REPORT vector to construct the wild-type SIRT7 luciferase reporter construct. The SIRT7 construct and miR-142-5p mimic or scrambled sequence were co-transfected into HK-2 cells by using Lipofectamine 2000. Lysates were harvested after 24 h, and the luciferase activity was measured using dual-luciferase assay. All procedures were performed in accordance with the manufacturer’s instructions.

### Hoechst staining

Cells were stained with Hoechst for 2 min to examine the typical apoptosis, including nuclei condensation and fragmentation. The fields were evaluated to determine the percentage of apoptosis.

### TdT-mediated dUTP nick end labeling (TUNEL) staining

In brief, kidney tissues were fixed with 4% paraformaldehyde and embedded with paraffin. Tissue sections of 4 μm were exposed to a TUNEL reaction mixture containing terminal deoxynucleotidyl transferase and nucleotides. Cell slides were fixed and the membrane was broken by the TUNEL reaction mixture, they were incubated at 4 °C overnight. Subsequently, the slides were examined by fluorescence microscopy.

### PCR

Total RNA was extracted using TRIzol according to the manufacturer’s instructions, and the A280 value of the sample was determined and quantified after purification. Exactly 2 μg total RNA was obtained, and reverse transcriptase was used to detect the relative expression of mRNA by real-time PCR. The PCR reaction system was operated under the following conditions: 1 μl of RT product, 10 μl of qPCR Mix and primer, and adjusted total volume of 25 μL by using double distilled water. The PCR reaction conditions were as follows: 95 °C for 5 min, 92 °C for 10 s, and 60 °C for 50 s for 40 cycles. The relative expression of mRNA was analyzed using 2^−ΔΔCt^ method. PCR primers were as follows: human-miR-142-5p: 5′-CTCAACTGGTGTCGTGGAGTCGGCAATTCAGTTGAGAGTAGTGC-3′ (forward), 5′-ACACTCCAGCTGGGCATAAAGTAGAAAGC-3′ (reverse); human-GAPDH:5′-GGAAGCTTGTCATCAATGGAAATC-3′ (sense), 5′-TGATGACCCTTTTGGCTCCC-3′ (antisense); mouse-miR-142-5p:5′-CTCAACTGGTGTCGTGGAGTCGGCAATTCAGTTGAGAGTAGTGC-3′ (forward), 5′-ACACTCCAGCTGGGCATAAAGTAGAAAGC-3′ (reverse); mouse-GAPDH:5′-CCTCGTCCCGTAGACAAAATG-3′ (sense), 5′-TGAGGTCAATGAAGGGGTCGT-3′ (antisense).

### Fluorescent miRNA *in-situ* hybridization

The fluorescent probe miR-142-5p was synthesized by Servicebio (Wuhan, China). In-situ hybridization was performed using the miR-142-5p probe in C57BL/6J mouse. Paraffin sections of mouse kidney tissue were dewaxed to water and digested with trypsin. After the sections were hybridized with oligonucleotide probes and incubated overnight at 37 °C in a thermostat, they were washed with sodium citrate solution (SSC) and then incubated with hybridization solution containing a dually labeled probe at 37 °C for 3 h. Subsequently, the sections were blocked, labeled with digoxigenin, stained with TSA, and re-stained with DAPI. Image acquisition and analysis were performed using an ortho-fluorescence microscope.

### Western blot

RIPA lysate was added to extract protein samples, and BCA protein quantitative kit was used for protein quantification. The loading amount of each group was adjusted, and four times of protein loading buffer solution was added. Denaturation was carried out in a water bath at 98° C for 5 min. After SDS-PAGE, the protein was transferred to PVDF membrane and sealed with 5% skimmed milk at room temperature for 90 min. Then different antibodies were added, incubated overnight at 4 °C, washed thrice with TBST for 5 min, added with anti-HRP labeled II antibody, and incubated at room temperature for 90 min. The antibodies were purchased from the following sources: Anti-SIRT7 (1:1,000, Wuhan, Boster), Anti-NF-κB (1: 1,000, Cell Signaling Technology), Anti-cleaved Caspase-3 (1:1,000, Affinity), P53 (1:1,000, Wuhan, Boster), Anti-GAPDH (1:1,000, Cell Signaling Technology). TBST was washed thrice for 10 min. The protein surface of PVDF membrane was immersed in ECL solution in a dark room to excite fluorescence. The protein bands were analyzed by gray scale analysis after pressing, developing, and fixing. The gray value of the electrophoresis strip was analyzed using the Quantity One software.

### Statistical analysis

Data were presented as the mean ± SD. The significance of differences between data was evaluated using one-way analysis of variance on SPSS 17.0 software, followed by Student–Newman–Kuels test. *p* < 0.05 was considered to represent a significant difference. All graphs shown in this manuscript were constructed using the GraphPad Prism 5.0 software.

## Results

### p53 was activated in kidney tubular epithelial cells after cisplatin treatment

**Figure 1.**
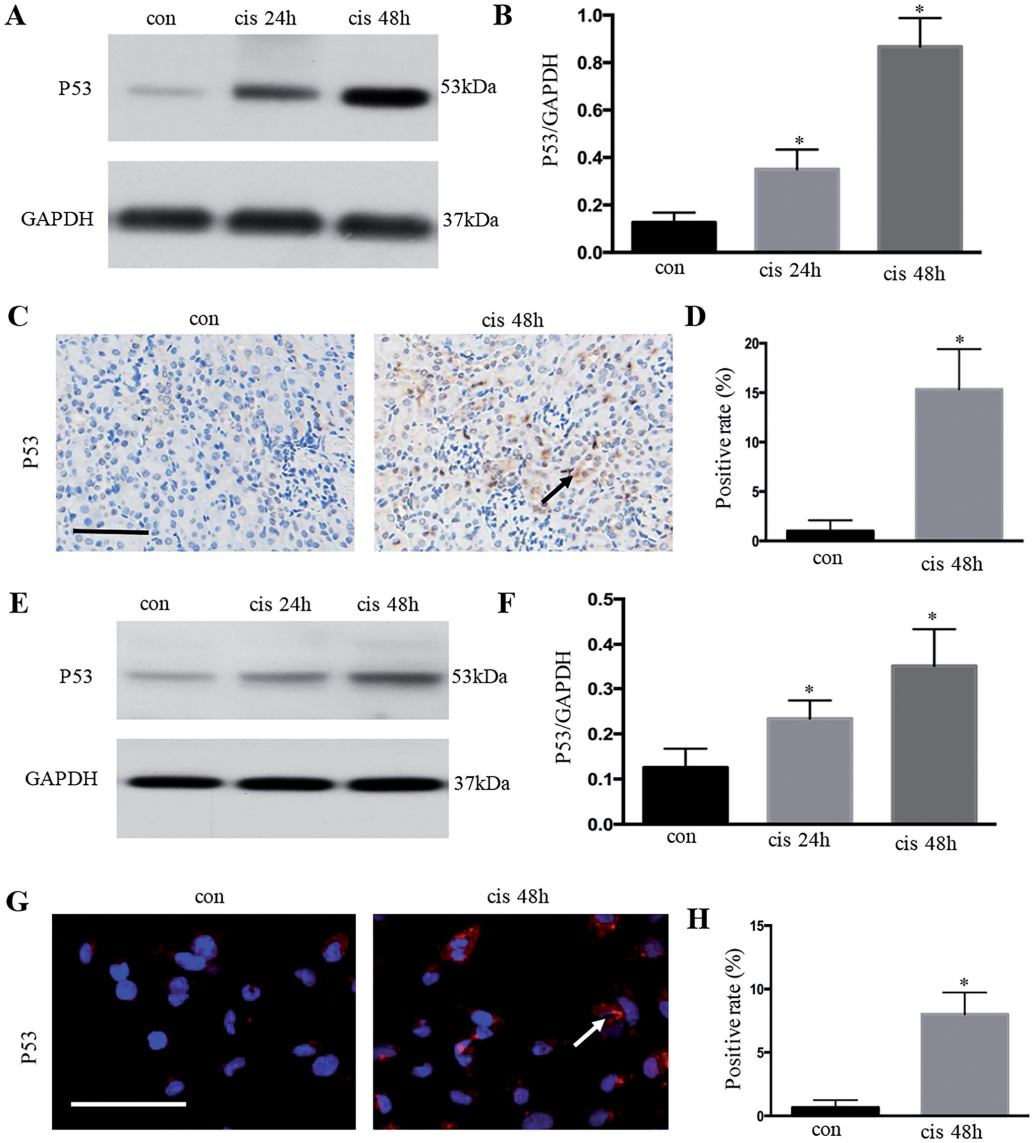
P53 was activated in kidney tubular epithelial cells after cisplatin treatment. (A) Expression of p53 in kidney tissue, as analyzed using Western blot analysis; (B) quantitative values of the relative expression levels of p53; (C) P53 expression (brown dots) of immunohistochemically stained kidney tissues from the control or cisplatin‐treated group; (D) percentage of positive cells in the control and cisplatin groups. Data were expressed as mean ± SD (*n* = 6); *p < 0.05 versus control. (E) Expression of p53 in HK-2 cells, as analyzed using Western blot analysis; (F) quantitative values of the relative expression levels of p53. G. p53 expression of immunofluorescence-stained HK-2 cells from the control or cisplatin‐treated group; (H) percentage of positive cells in the control and cisplatin-treated group. Data were expressed as mean ± SD (*n* = 3); *p < 0.05 versus control.

**Figure 2.**
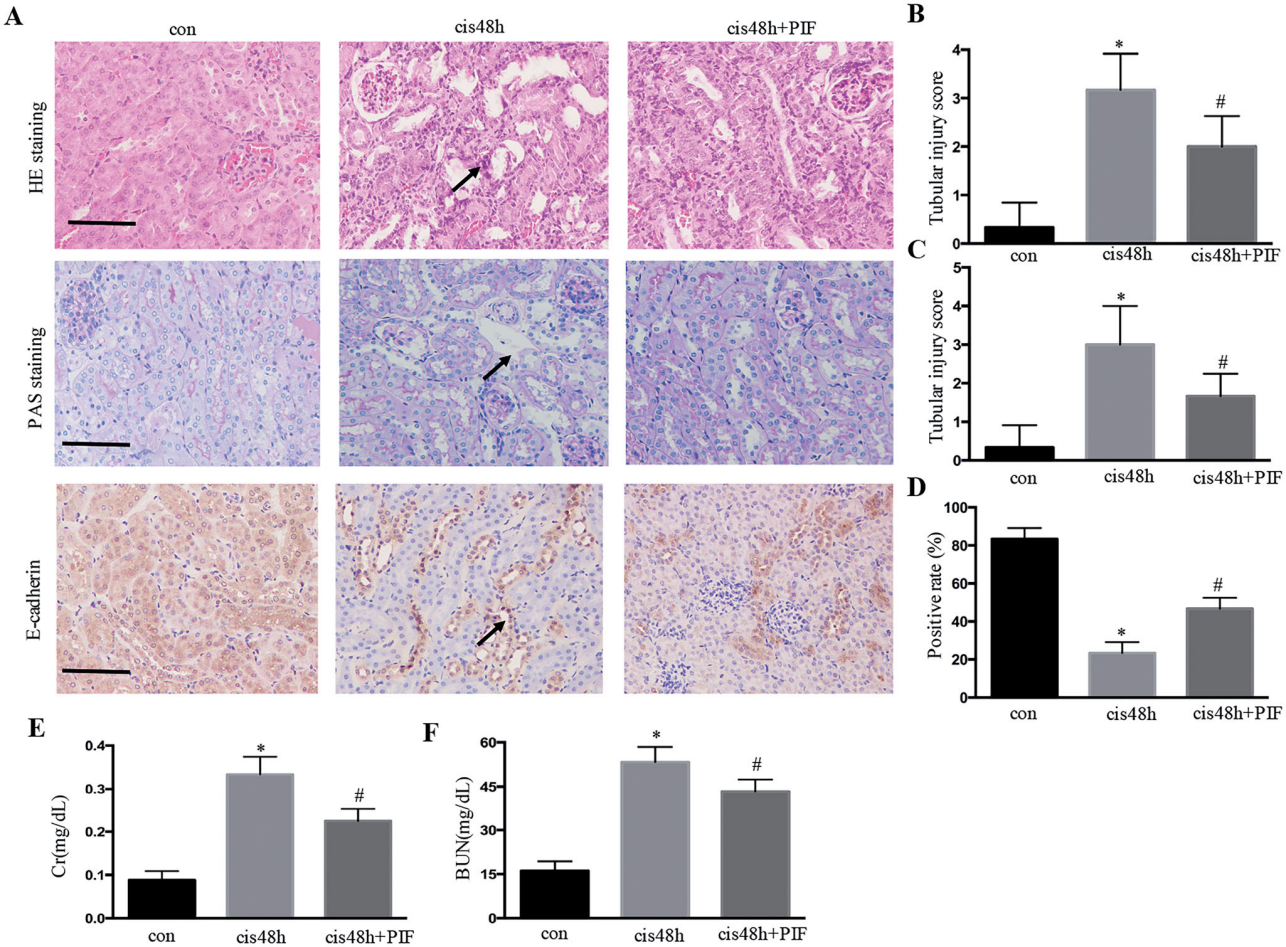
Inhibition of p53 attenuated renal histological and functional damage after cisplatin treatment. (A) HE, PAS, and E-cadherin immunohistochemical staining for the analysis of renal injury in the kidney; (B) tubular damage score for HE staining; (C) tubular damage score for PAS staining; (D) quantification of E-cadherin; (E and F) Cr and BUN levels. Data were expressed as mean ± SD (*n* = 6); *p < 0.05 versus control, ^#^*p* < 0.05 versus cisplatin.

The p53 signaling pathway is involved in cisplatin-induced acute kidney injury (AKI). Western blot analysis showed that p53 was activated in the kidney (Figures 1(A,B)) and in HK-2 cells (Figures 1(E), 2(F)) from 24 h to 48 h with cisplatin treatment. Immunohistochemical and immunofluorescent staining showed that the accumulation of p53 was mainly in the nucleus of renal tubular epithelium after cisplatin treatment for 48 h, and the expression level was significantly higher than that in the control group (Figures 1(C–H)).

### Inhibition of p53 attenuated renal histological and functional damage after cisplatin treatment

Pifithrin-α is a pharmacological inhibitor of p53. The effects of pifithrin-α in mice were initially examined to determine the role of p53 in cisplatin-induced AKI *in vivo*. As shown in Figures 2(A–D), cisplatin treatment induced obvious kidney tubular injury for 48 h, and pifithrin-α treatment partially inhibited renal histological damage, as demonstrated by HE and PAS staining. Importantly, pifithrin‐α treatment also improved renal function, and the BUN and serum creatinine in pifithrin‐α-treated animals decreased compared with those in the cisplatin-treated group (Figures 2(E,F)).

### Inhibition of p53 attenuated renal tubular apoptosis after cisplatin treatment *in vivo* and *in vitro*

**Figure 3.**
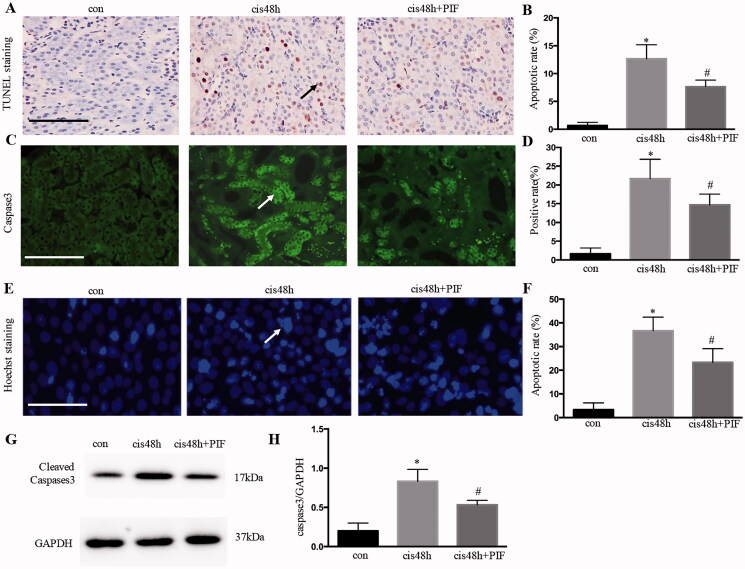
Inhibition of p53 attenuated renal tubular apoptosis after cisplatin treatment *in vivo* and *in vitro*. (A) TUNEL staining and immunofluorescence of caspase-3 for the analysis of renal tubular apoptosis in the kidney; (B) apoptotic rate of cells examined using TUNEL staining; (C) quantification of caspase-3. Data were expressed as mean ± SD (*n* = 6); *p < 0.05 versus control, ^#^*p* < 0.05 versus cisplatin. (D) Apoptosis of cells examined using Hoechs; (E) apoptotic ratio of renal tubular cells; (F) immunoblotting analysis of caspase-3; (G) quantification of caspase-3 by densitometry. Data were expressed as mean ± SD (*n* = 3); *p < 0.05 versus control, ^#^*p* < 0.05 versus cisplatin.

TUNEL assay indicated that cisplatin treatment induced renal tubular apoptosis for 48 h was markedly attenuated in pifithrin‐α-treated animals (Figures 3(A,B)). Consistently, caspase activation, as shown by caspase-3 immunofluorescence, was also decreased in pifithrin‐α-treated animals (Figures 3(C,D)). Moreover, pifithrin‐α treatment extensively reduced HK-2 cell apoptosis compared with cisplatin treatment for 48 h (Figures 3(E,F)). Western blot analysis further validated that the cellular expression of apoptosis-related protein caspase-3 was significantly increased after cisplatin treatment for 48 h, whereas the inhibition of p53 decreased the expression of caspase-3 (Figures 3(G,H)). Together, these pharmacological results supported the protective role of p53 inhibition in cisplatin-induced AKI *in vivo* and *vitro*.

### MiR-142-5p was downregulated in cisplatin-induced kidney tissues

**Figure 4.**
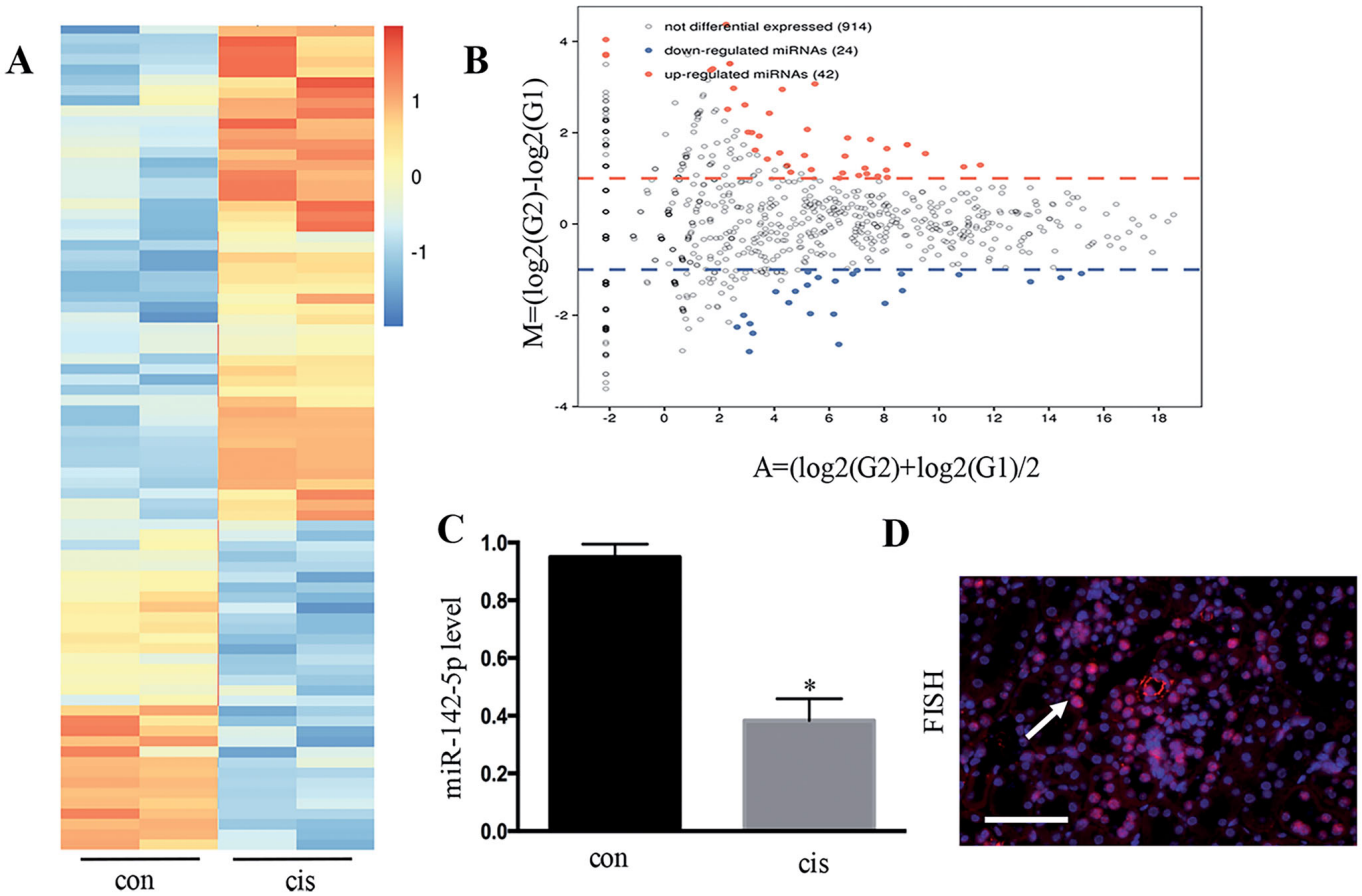
MiR-142-5p was downregulated in cisplatin-induced kidney tissues. (A) MicroRNA expression, as determined by microarray analysis after cisplatin-induced kidney injury in mice; (B) MA plot showing 42 microRNAs with significantly increased changes in expression and 24 microRNAs with significantly decreased changes in expression following cisplatin treatment; (C) PCR analysis for the detection of the fold-change of miR-142-5p from cisplatin-treated mouse kidneys; (D) FISH analysis of miR-142-5p during cisplatin treatment of HK-2 cells. Data were expressed as mean ± SD (*n* = 3); *p < 0.05 versus control.

The miRNA expression in cisplatin-induced AKI in mice was analyzed using microarray analysis (Figure 4(A)). A total of 42 miRNAs showed remarkably increased expression, whereas 24 miRNAs showed remarkably decreased expression following cisplatin treatment (Figure 4(B)). In the downregulated miRNAs, the induction of miR-142-5p was confirmed using real-time PCR (Figure 4(C)). In addition, miR-142-5p was distributed in the nuclei and cytoplasm by fluorescence in-situ hybridization (Figure 4(D)).

### Overexpression of miR‐142‐5p reduced HK‐2 cell apoptosis during cisplatin treatment

**Figure 5.**
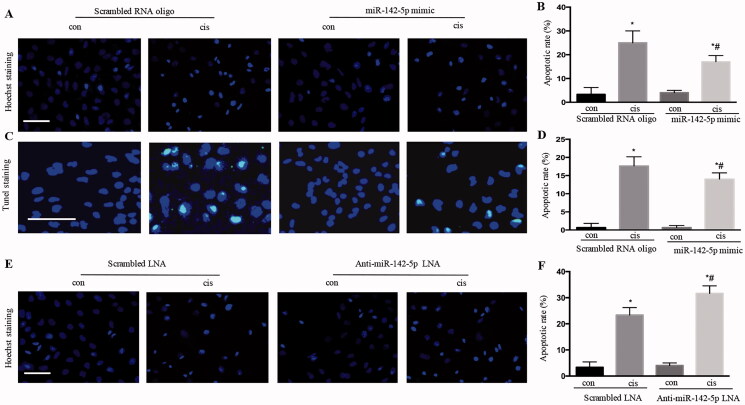
Overexpression of miR-142-5p-reduced HK‐2 cell apoptosis during cisplatin treatment. (A) typical apoptotic nucleus in cells, as revealed by Hoechst staining after miR‐142‐5p mimic treatment; (B) apoptotic ratio of renal tubular cells; (C) apoptotic nucleus in cells, as revealed by TUNEL staining after miR‐142‐5p mimic treatment; (D) apoptotic ratio of renal tubular cells; (E) typical apoptotic nucleus in cells, as revealed by Hoechst staining after anti-miR‐142‐5p-LNA treatment; (F) apoptotic ratio of renal tubular cells. Data were expressed as mean ± SD (*n* = 3); *p < 0.05 versus control, ^#^*p* < 0.05 versus cisplatin.

As shown in Figure 5(A), the HK-2 cells showed typical apoptotic nucleus in Hoechst staining. Overexpression of miR‐142‐5p by miR-142-5p mimic remarkably reduced HK-2 cell apoptosis during cisplatin treatment for 24 h, as revealed by Hoechst staining and TUNEL staining (Figures 5(B–D)). Instead, the inhibition of miR-142-5p by anti-miR-142-5p LNA significantly increased the HK-2 cell apoptosis (Figures 5(E,F)). Together, these results suggested that miR-142-5p may contribute to tubular cell injury during cisplatin-induced AKI.

### P53 inhibition attenuated cisplatin-induced AKI by upregulating microRNA-142-5p

**Figure 6.**
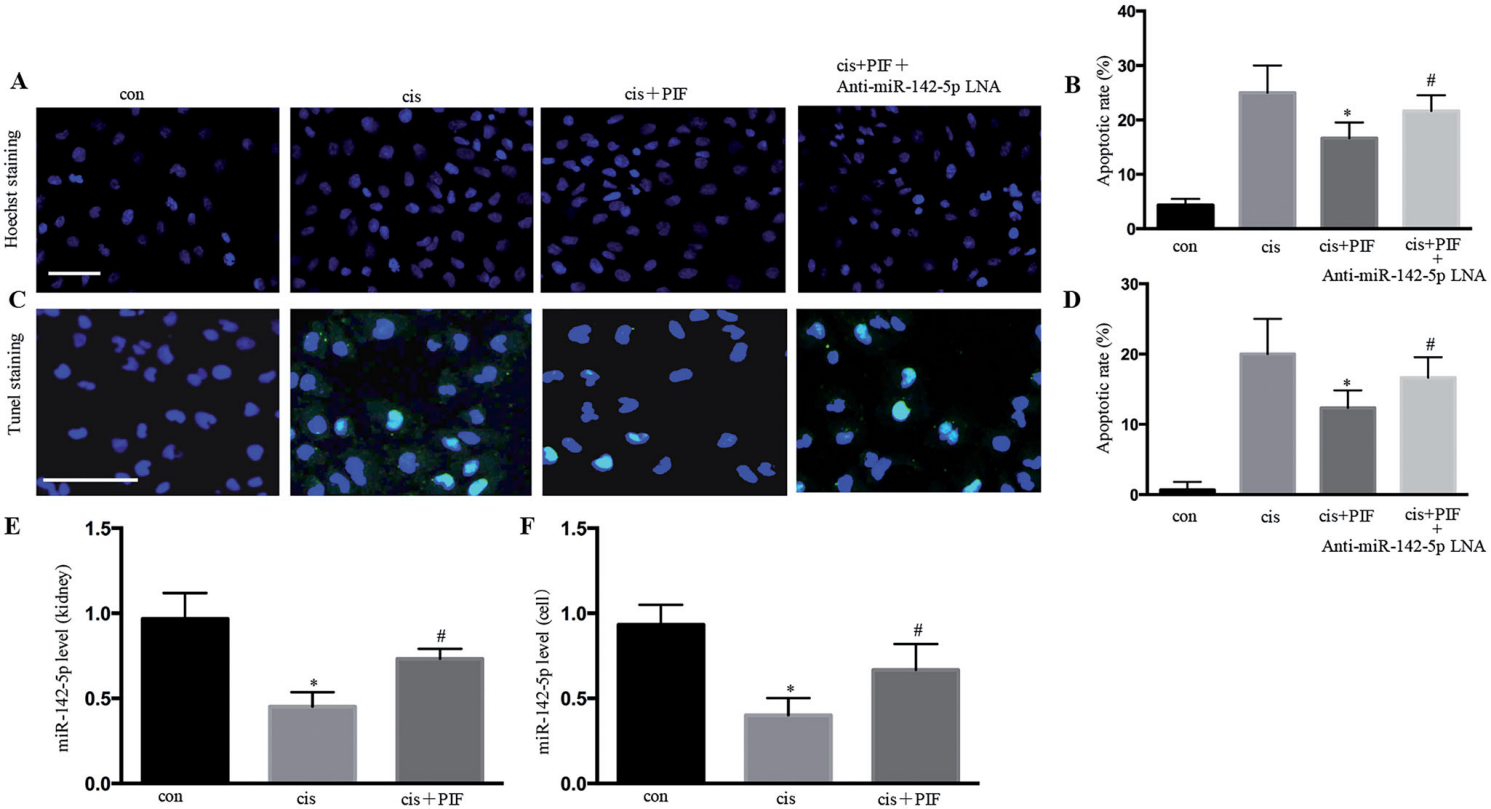
P53 inhibition attenuated cisplatin-induced acute kidney injury by upregulating microRNA-142-5p. (A) Typical apoptotic nucleus in cells, as revealed by Hoechst staining; (B) apoptotic ratio of renal tubular cells; (C) apoptotic nucleus in cells, as revealed by TUNEL staining; (D) apoptotic ratio of renal tubulacells; (E) detection of miR‐142‐5p expression by RT-PCR in AKI mice; (F) Detection of miR‐142‐5p expression by RT-PCR in HK-2 cells. Data were expressed as mean ± SD (*n* = 3); *p < 0.05 versus control, ^#^*p* < 0.05 versus cisplatin.

Hoechst staining showed that the apoptotic rate was higher in pifithrin-α- and anti-miR-142-5p-LNA-interfered cisplatin-treated renal tubular epithelial cells than that in pifithrin-α- and cisplatin-treated renal tubular epithelial cells (Figures 6(A,B)). TUNEL staining showed that the apoptotic rate was higher in pifithrin-α- and anti-miR-142-5p-LNA-interfered cisplatin-treated renal tubular epithelial cells than that in pifithrin-α and cisplatin-treated renal tubular epithelial cells (Figures 6(C,D)). The miR‐142‐5p expression was detected using RT-PCR, and the results suggested that pifithrin‐α upregulated miR‐142‐5p in the kidney and HK-2 cells with cisplatin treatment (Figures 6(E,F)). Together, these results suggested that p53 inhibition attenuated cisplatin-induced AKI by upregulating microRNA-142-5p.

### MiR-142-5p targeted SIRT7 during cisplatin treatment

**Figure 7.**
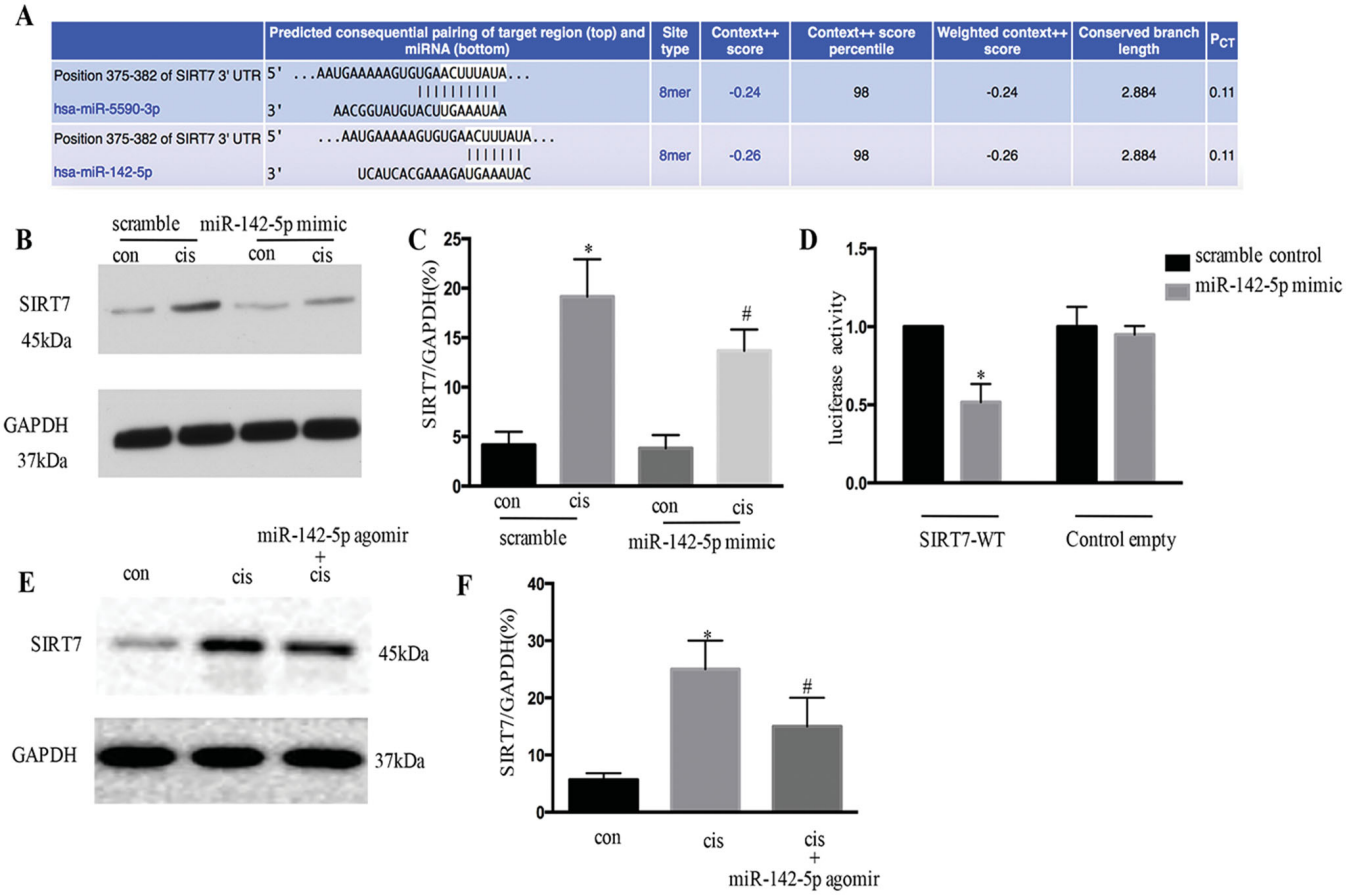
MiR-142-5p targets SIRT7 during cisplatin treatment in HK-2 cells. (A) Conserved putative miR-142-5p targeting site in the 3′ UTR of SIRT7 mRNA, as identified using online databases (TargetScan); (B) immunoblotting analysis of SIRT7 protein expression levels; (C) quantitative analysis of the expression of SIRT7 (*p < 0.05 versus scramble, ^#^*p* < 0.05 versus cisplatin); (D) double luciferase assay carried out to confirm the target gene of miR-142-5p after co-transfection of SIRT7 3′ UTR double luciferase reporter gene vector and miR-142-5p mimics; (E) SIRT7 level examined by Western blot after miR-142-5p treatment in mice induced by cisplatin; F. quantification of SIRT7 by densitometry. Data were expressed as mean ± SD (*n* = 3); *p < 0.05 versus control, ^#^*p* < 0.05 versus cisplatin.

The potential target of miR-142-5p was identified as SIRT7 by TargetScan (Figure 7(A)). The overexpression of miR-142-5p significantly repressed SIRT7 in cisplatin-treated HK-2 cells (Figures 7(B,C)). Moreover, miR-142-5p mimics suppressed the luciferase activity in SIRT7-miR-142-5P-transfected cells (Figure 7(D)). In this cisplatin animal model, the miR-142-5p-treated mice consistently showed a decrease in SIRT7 expression after cisplatin induced AKI (Figures 7(E,F)). Overall, these results suggested that SIRT7 was a target gene of miR-142-5p.

### SIRT7 inhibition reduced HK‐2 cells apoptosis with cisplatin treatment

**Figure 8.**
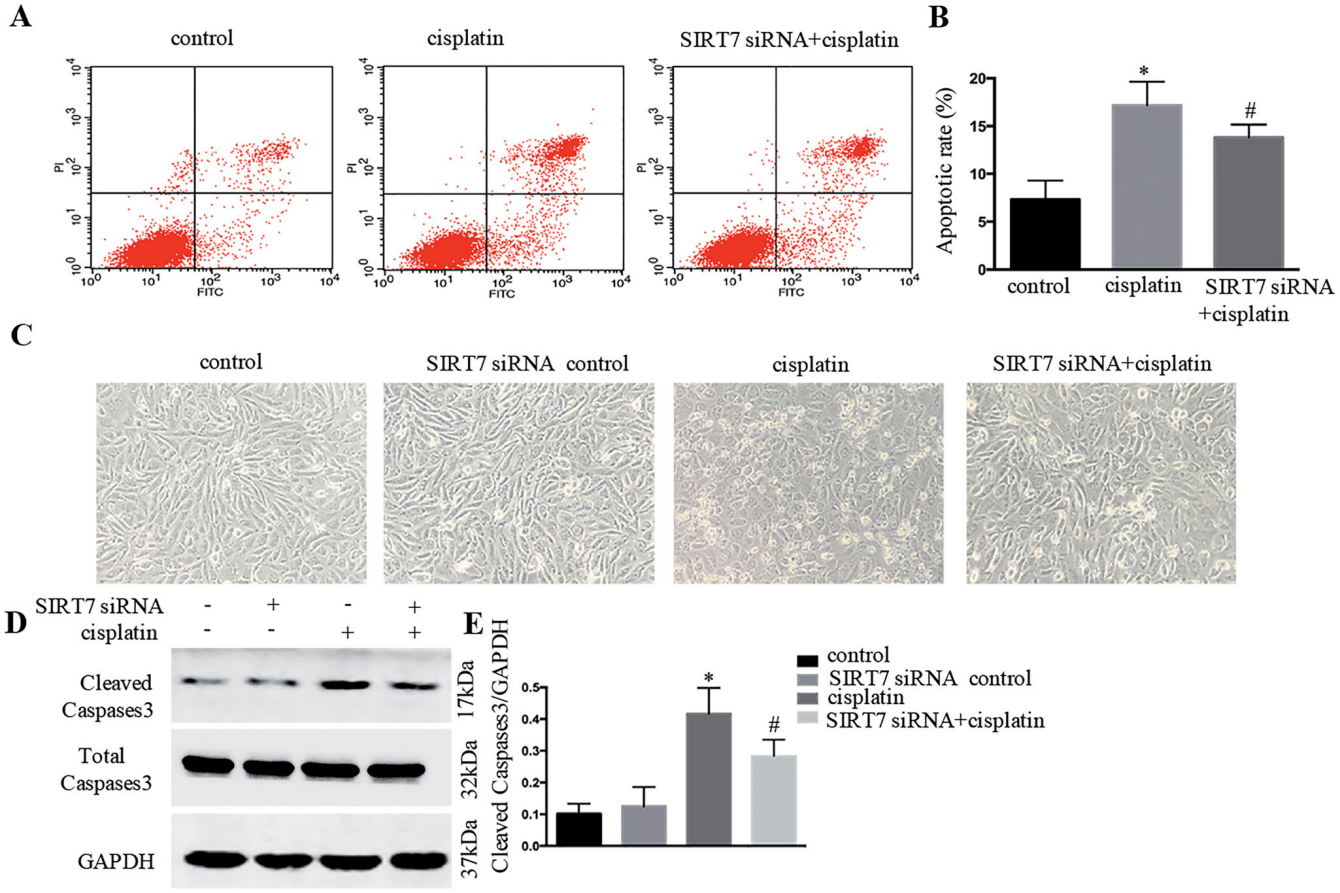
SIRT7 inhibition reduced HK‐2 cell apoptosis with cisplatin treatment. (A) Apoptotic cells revealed by flow cytometry; (B) apoptotic ratio of renal tubular cells; (C) HK-2 cells apoptosis observed by morphology; (D) immunoblotting analysis of caspase-3; (E) quantification of caspase-3 by densitometry. Data were expressed as mean ± SD (*n* = 3); *p < 0.05 versus control, ^#^*p* < 0.05 versus cisplatin.

As shown in Figures 8(A,B), flow cytometry analyses revealed that the number of apoptotic cells in the siRNA SIRT7 group was lower than that in the cisplatin group. SiRNA SIRT7 treatment significantly inhibited apoptosis, as shown in morphological observation (Figure 8(C)). Moreover, siRNA SIRT7 treatment remarkably suppressed caspase-3 processing/activation based on immunoblot analysis (Figures 8(D,E)).

### NF-κB pathway was involved in the p53/microRNA-142-5p/SIRT7- attenuated cisplatin-induced AKI

**Figure 9.**
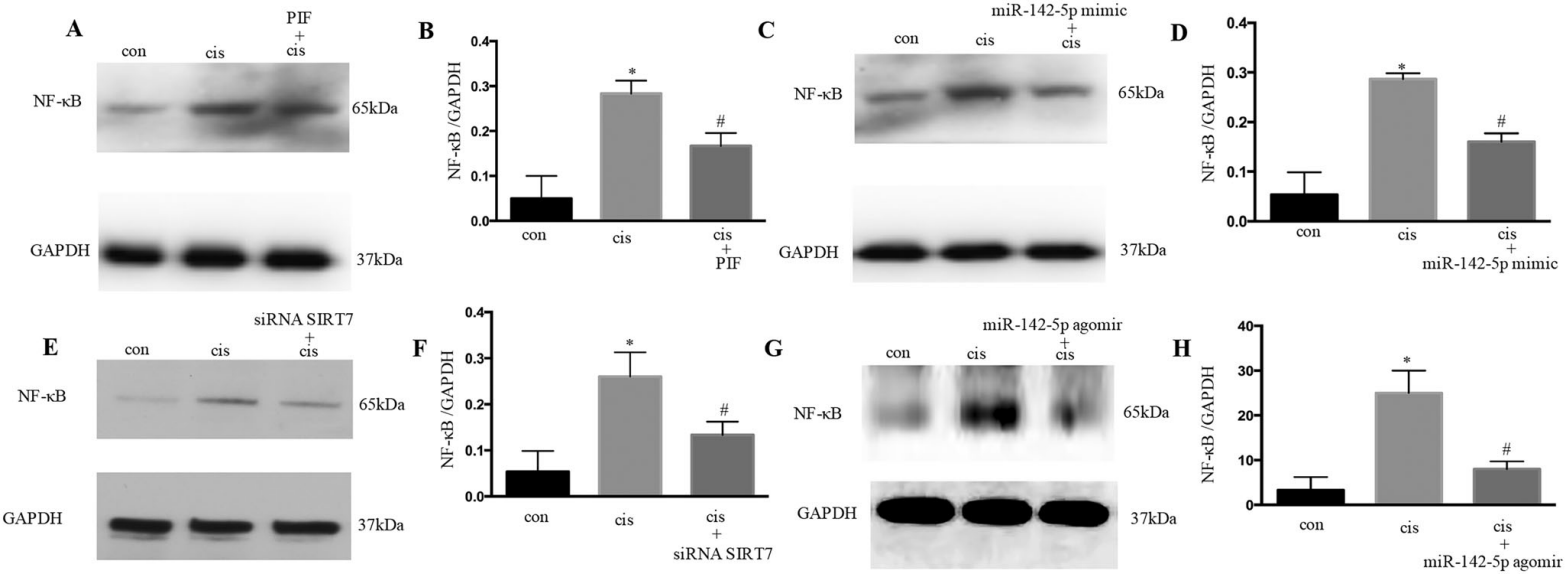
NF-κB pathway was involved in the p53/miR-142-5p/SIRT7 attenuation of cisplatin-induced acute kidney injury. (A) Immunoblotting analysis of NF-κB after pifithrin‐α treatment; (B) quantification of NF-κB by densitometry; (C) immunoblotting analysis of NF-κB after miR-142-5p mimic treatment; (D) quantification of NF-κB by densitometry; (E) immunoblotting analysis of NF-κB after siRNA SIRT7 treatment; (F) quantification of NF-κB by densitometry; (G) NF-κB level examined by Western blot after miR-142-5p treatment in mice induced by cisplatin; (H) quantification of NF-κB by densitometry. Data were expressed as mean ± SD (*n* = 3); *p < 0.05 versus control, ^#^*p* < 0.05 versus cisplatin.

NF-κB plays an important role in cisplatin-induced AKI. The results showed that pifithrin-α treatment could suppress NF-κB during cisplatin treatment (Figures 9(A,B)). In addition, the overexpression of miR-142-5p significantly repressed NF-κB in HK-2 cells (Figures 9(C,D)). The inhibition of SIRT7 also remarkably suppressed NF-κB based on immunoblot analysis (Figures 9(E,F)). In this cisplatin animal model, the miR-142-5p-treated mice consistently showed a decrease in NF-κB expression after cisplatin induced AKI (Figures 9(G,H)). Overall, these results suggested that p53/microRNA-142-5p/SIRT7 attenuated cisplatin-induced AKI through NF-κB.

## Discussion

In this study, miR-142-5p was found to be one of the most highly expressed miRNAs in renal tubular epithelial cells, and its expression was substantially downregulated in the cisplatin-induced renal injury model and HK-2 apoptosis model. This parameter was positively correlated with the degree of apoptosis.

MiRNAs are a class of endogenous non-coding RNA molecules that play an important role in regulating gene expression at the post-transcriptional level. Recent research has demonstrated that miRNAs play an important role in cisplatin nephrotoxicity and cisplatin-induced renal tubular cell apoptosis [18,19]. MiR-155 regulates the cisplatin-induced apoptosis of renal tubular epithelial cells by targeting c-Fos [20]. Huang *et al.* showed the involvement of miR-181a/PTEN axis in the renoprotective effect of curcumin against cisplatin-induced AKI, and provide new evidence on the ability of curcumin to alleviate cisplatin-induced nephrotoxicity [21]. The present study focused on miR-142-5p, because its induction was verified in kidney and renal tubular epithelial cells with cisplatin treatment. MiR-142-5p plays important roles in several cancers and non-cancerous diseases [22,23]. It enhances cisplatin-induced apoptosis in ovarian cancer cells by targeting multiple anti-apoptotic genes [24]. It also regulates pancreatic cancer cell proliferation and apoptosis by regulating RAP1A [25]. Furthermore, miR-142-5p is a biomarker for the early detection of chronic antibody-mediated rejection in kidney transplantation [26]. The overexpression of miR-142-5p during hypoxia induces extensive cell injury and apoptosis, whereas its suppression substantially promotes cardiomyoblast and primary cardiomyocyte cell viability and attenuates cell apoptosis with hypoxia treatment [27]. In the present study, the role of miR-142-5p in the apoptotic response of renal tubular cells was determined.

P53 plays an important role in cisplatin nephrotoxicity, and it may regulate many non-coding genes, including miRNAs [28,29]. As a transcription factor, p53 plays its role through the transcription regulation of its target gene and starts many cell responses [30]. P53 also participates in the process of cisplatin-induced acute renal injury, and its inhibition could reduce acute renal injury [31,32]. In the present study, the inhibition of p53 expression substantially reduced the cisplatin-induced apoptosis of renal tubular epithelial cells, and these findings are consistent with most of the reported results. Several p53-miRNA networks have also been studied in cisplatin nephrotoxicity [33,34]. In the present study, the inhibition of p53 remarkably increased the miR-142-5p expression in kidney and renal tubular cells after cisplatin treatment.

The miR‐142‐5p downstream target genes were searched using TargetScan, and a binding site at the 3′ UTR of SIRT7 was predicted on miR-142-5P. After co-transfection of the SIRT7 3′ UTR double luciferase reporter gene vector and miR‐142‐5p mimics, double luciferase assay was carried out to confirm that the target gene of miR‐142‐5p was SIRT7. The overexpression of miR-142-5p remarkably decreased SIRT7 expression. SIRT7 plays an important role in oncogenic transformation [35]. Li *et al.* have proven that SIRT7 knockdown inhibits the proliferation and cell cycle of HUCCT1 cells [36]. SIRT7 is involved in the regulation of cell apoptosis and stress response in the heart [37]. Similarly, based on these findings, SIRT7 deficiency ameliorates cisplatin-induced AKI through the regulation of the inflammatory response [38]. SIRTs regulate NF-κB in various manners [39]. SIRT7 deficiency suppresses the nuclear accumulation of p65, which is a component of NF-κB [40]. Moreover, SIRT7 knockdown promotes the translocation of NF-κB p-p65 to the cell nucleus and then increases the secretion of inflammatory cytokines. SIRT7 could suppress LPS-induced inflammation and apoptosis *via* the NF-κB signaling pathway [41]. NF-κB is an important transcription factor involved in inflammatory response, cell proliferation, and apoptotic processes in cells [42]. When cells are stimulated by multiple factors (such as bacterial infection, oxidative stress, and antigenic immunity), IκBα is phosphorylated and rapidly degraded, and then NF-κB translocates to the nucleus to start transcriptional regulation, promoting the expression of key downstream inflammatory factors, such as TNF-α, IL-1β and IL-6; in turn, it initiates an inflammatory response [43]. In the present study, the results showed that SIRT7 knockdown suppressed cisplatin-induced apoptosis *via* the NF-κB signaling pathway.

**Figure 10.**
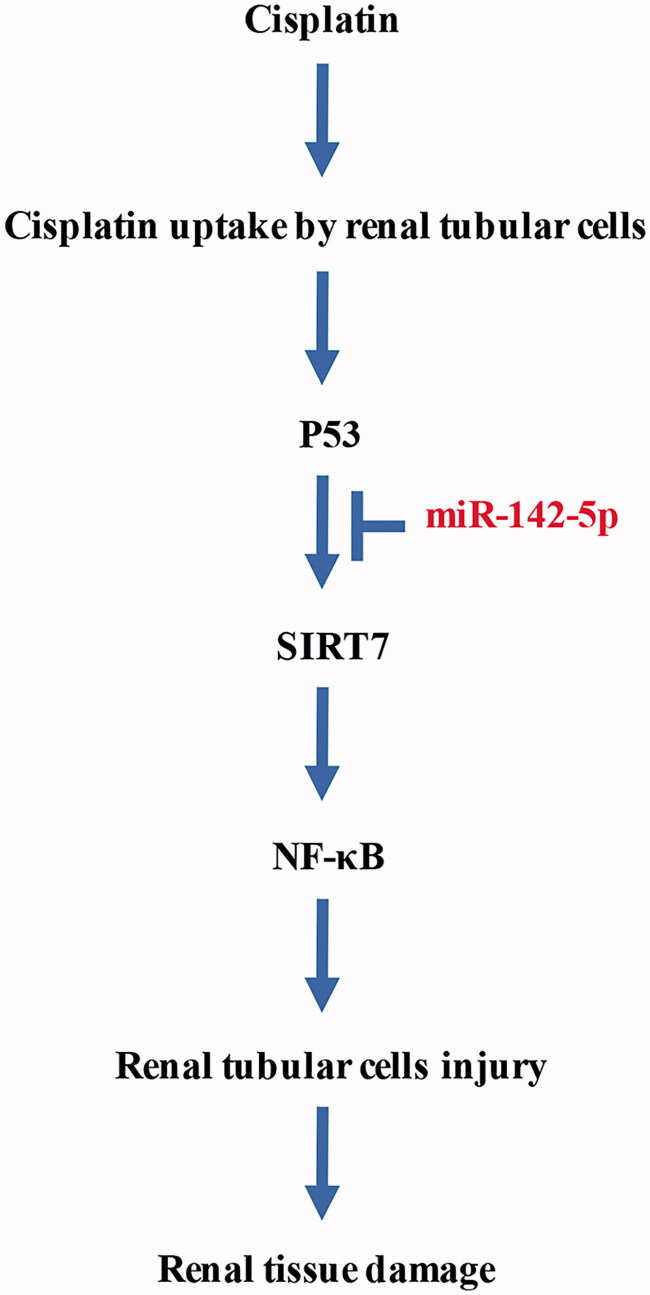
P53/miR-142-5p/SIRT7/NF-κB pathway in cisplatin-induced acute kidney injury.

In conclusion, the overexpression of miR-142-5p could alleviate the cisplatin-induced acute injury of renal tubular epithelial cells, and this phenomenon is possibly related to the inhibition of cell apoptosis. The p53/miR-142-5P/SIRT7/NF-κB pathway may become a new target for the diagnosis and treatment of cisplatin-induced AKI (Figure 10).
